# What factor within the Japanese Association for Acute Medicine (JAAM) disseminated intravascular coagulation (DIC) criteria is most strongly correlated with trauma induced DIC? A retrospective study using thromboelastometry in a single center in Japan

**DOI:** 10.1007/s00068-016-0756-4

**Published:** 2017-01-16

**Authors:** H. Koami, Y. Sakamoto, K. C. Yamada, T. Matsuda, J. Nishi, K. Nakayama, R. Sakurai, M. Ohta, H. Imahase, M. Yahata, M. Umeka, T. Miike, F. Nagashima, T. Iwamura, S. Inoue

**Affiliations:** 1 Department of Emergency and Critical Care Medicine, Faculty of Medicine, Saga University, 5-1-1, Nabeshima, Saga City, Saga 8498501 Japan; 2 Advanced Emergency Care Center, Saga University Hospital, 5-1-1, Nabeshima, Saga City, Saga 8498501 Japan; 3 Division of Trauma Surgery and Surgical Critical Care, Faculty of Medicine, Saga University, 5-1-1, Nabeshima, Saga City, Saga 8498501 Japan

**Keywords:** Trauma, DIC, JAAM DIC score, PT-INR, Thromboelastometry

## Abstract

**Purpose:**

The diagnostic criteria for disseminated intravascular coagulation (DIC) established by the Japanese Association for Acute Medicine (JAAM) is able to diagnose DIC accurately and promptly. The aim of this retrospective study is to evaluate the degree of association between each parameter of JAAM DIC criteria and the diagnosis of trauma induced DIC (T-DIC) utilizing thromboelastometry (ROTEM).

**Methods:**

Trauma patients transported to our hospital with ROTEM performed in the emergency department between January 2013 and December 2015 were enrolled in this study. We evaluated (1) the characteristics of T-DIC, (2) the relationships between T-DIC and each parameter of the JAAM DIC criteria and (3) the diagnostic accuracies of each parameter for T-DIC by statistical measurement.

**Results:**

All 72 patients (21 T-DIC and 51 control) were included in primary analysis. T-DIC was significantly related to younger age, more severe trauma scores, more cases of massive transfusions, and remarkable coagulation abnormality detected by standard coagulation tests. In the cases of T-DIC, ROTEM showed longer clotting time, lower acceleration, lower clot firmness, and inhibited fibrinolysis in EXTEM/INTEM. Within the JAAM DIC score, PT-INR ≥1.2 was the most accurate factor for T-DIC diagnosis; sensitivity 60.0%, specificity 100.0%, and accuracy 88.7%. PT-INR ≥1.2 was statistically correlated with the JAAM DIC score (*p* < 0.001, *r* = 0.709). The univariate analysis based on 1.2 of PT-INR indicated statistical differences in most categories of ROTEM, which is similar to analysis performed for the presence and absence of T-DIC.

**Conclusions:**

Among JAAM DIC criteria, the PT-INR ≥1.2 was the most accurate factor for both the diagnosis of T-DIC and the evaluation of its severity.

## Introduction

Trauma is a leading cause of death and disability worldwide [1, 2]. The pathophysiological features of trauma are affected by various factors including type of injury source, underlying medical conditions, demographics of the victim, quality of the initial trauma care at the scene, and the strategy of advanced trauma resuscitation in a trauma center [3–8].

Bleeding is a primary cause of trauma death, and coagulopathy is strongly associated with an increased requirement for blood transfusions and poor clinical outcome [9–11]. Recent articles report that disseminated intravascular coagulation (DIC) plays a pivotal role in the pathogenesis of post-traumatic organ dysfunction in severe trauma patients [12]. However, it is necessary to determine the hematological status promptly and correctly to initiate effective hematological resuscitations in the emergency department (ED). Scoring systems utilizing several biomarkers were developed by the Japanese Ministry of Health and Welfare (JMHW) in 1987 and the International Society of Thrombosis and Homeostasis (JSTH) in 2001 [13, 14]. Although these criteria had potential to accurately diagnose DIC in critically ill patients, some practical limitations were stated [15, 16]. Thus, new diagnostic criteria were established by the Japanese Association for Acute Medicine (JAAM) in 2006 [17]. These criteria were proven to be able to diagnose DIC more accurately and select patients were able to receive resuscitation at the early phase of DIC compared with former criteria [17, 18].

Rotational thromboelastometry (ROTEM; TEM International, GmbH, Munich, Germany) is known as a point-of-care viscoelastic test using a citrated whole-blood sample. This test can detect coagulation and fibrinolytic abnormalities more rapidly and pathophysiologically than standard coagulation tests [19]. To date, more than 1000 articles about ROTEM have been published in various fields including cardiovascular surgery, liver transplantation, and trauma surgery [20–22].

The JAAM DIC criteria, which were established based upon all possible causes of critically ill patients, consist of widely available biomarkers and vital signs that can be measured in the emergency room [17]. However, few studies have reported about the level of contribution of each parameter within these criteria to the diagnosis of DIC. Further, T-DIC should be differentiated from non-trauma related DIC, because each DIC patient with different etiology tends to have a wide variety of clinical manifestations and mechanisms of coagulopathy.

The aim of this retrospective study is to elucidate the most meaningful and relevant parameter of the JAAM DIC criteria to the diagnosis of trauma induced DIC (T-DIC).

## Methods

### Patients and study design

This retrospective study has been approved by the institutional review board of Saga University Hospital (Protocol Identification Number: 2014-09-08). Trauma patients who were transported to our hospital and had ROTEM performed in the emergency department (ED) between January 2013 and December 2015 were enrolled. Patients with out-of-hospital cardiac arrest, burn injury, electrical injury, less than 18 years of age, or shorter length of hospital stay (<2 days) were excluded. The enrolled patients were divided into two groups based on the presence or absence of DIC. Univariate analysis was performed to evaluate the hematological characteristics of T-DIC, the relationships between T-DIC and each parameter of the JAAM DIC criteria, and the diagnostic accuracies of each parameter for diagnosing T-DIC. We further evaluated the differences in ROTEM data in accordance with each factor of the JAAM criteria. The correlation between the JAAM DIC score and the most accurate parameter in the criteria was also evaluated.

### Diagnosis of DIC and other clinical parameters

**Fig. 1:**
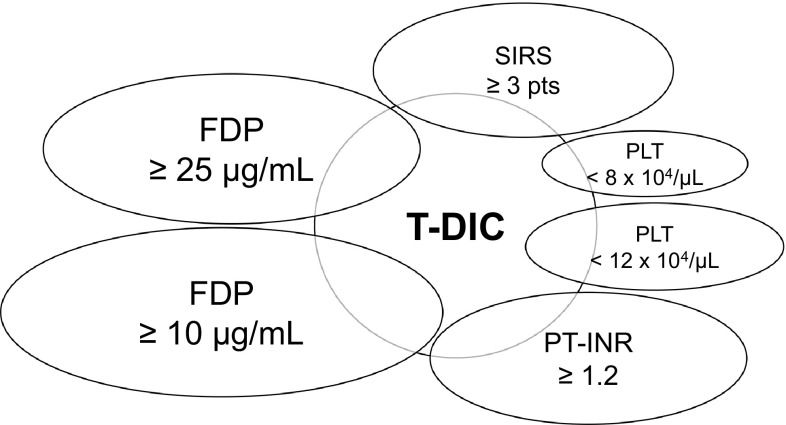
Scheme of this study

All trauma patients were diagnosed with DIC by the JAAM DIC criteria on their admission to the ED [17]. DIC was defined when the total score was 4 points or greater (range 0–8) by the criteria. We utilized the parameters of the JAAM DIC criteria for subsequent analyses including (1) systemic inflammatory response syndrome (SIRS) ≥3 pts, (2) platelet (PLT) <8 × 10^4^/µL or (3) PLT <12 × 10^4^/µL, (4) international normalized ratio of prothrombin time (PT-INR) ≥1.2, (5) fibrinogen and fibrin degradation products (FDP) ≥25 µg/mL or (6) FDP ≥10 µg/mL (Fig. 1). Patient characteristics and past medical histories were evaluated. The definition of shock was a systolic blood pressure of less than 90 mmHg on admission. Various trauma scores including the injury severity score (ISS), the revised trauma score (RTS) and the probability of survival (Ps) were evaluated from the medical records, retrospectively. Massive transfusion was defined to be more than 10 units of red blood cell (RCC-LR) transfusion required within the first 24 h of admission to the ED. Emergency surgery was performed for hemostasis within the first 24 h.

### Laboratory tests

Blood samples were collected by an emergency physician immediately after admission to the ED. Laboratory data [white blood cell (WBC), hemoglobin (Hb), PLT, PT-INR, activated partial thromboplastin time (APTT), fibrinogen, FDP, d-dimer (DD) and lactate] were measured in all trauma patients.

### ROTEM analysis

To determine the coagulation and fibrinolytic status, ROTEM was often performed for trauma patients in our department. Our thromboelastometric analysis was focused on four assays: extrinsic coagulation cascade (EXTEM), intrinsic coagulation cascade (INTEM), function of fibrinogen (FIBTEM) and anti-fibrinolytic cascade using aprotinin (APTEM). ROTEM parameters analyzed in this study included the clotting time (CT), the clot formation time (CFT), the alpha angle (α), the amplitude at 10 min (A10), 20 min (A20), and 30 min (A30), the maximum clot firmness (MCF), the lysis index at 30 min (LI30), and maximum lysis (ML). These were classified into four main categories according to their features: (1) initiation (CT), (2) acceleration (CFT, α), (3) clot firmness (A10, A20, A30, MCF), and (4) fibrinolysis (LI30, ML). Hyperfibrinolysis was defined by 20% improvement of fibrinolysis in the APTEM test compared with the EXTEM test, when the ML of EXTEM was 15% or more. Physician in charge selected patients who needed to take ROTEM analysis. All tests were started less than 1 h after admission to the ED and ran more than 60 min at 37 °C.

### Statistical analysis

All continuous variables are represented as median [interquartile range (IQR); Q1–Q3] and categorical variables as numbers (percentages). The *p* values were calculated from the Mann–Whitney *U* test for continuous variables, and Fisher’s exact test and Chi-square tests were used for categorical variables. Spearman correlation analysis and curve fitting were used to evaluate the relationship between the JAAM DIC score and a statistically significant parameter. Values of *p* < 0.05 were considered to be significant. Statistical analyses were performed by IBM SPSS Statistics version 23 (IBM Corp., Armonk, NY, USA).

## Results

**Fig. 2:**
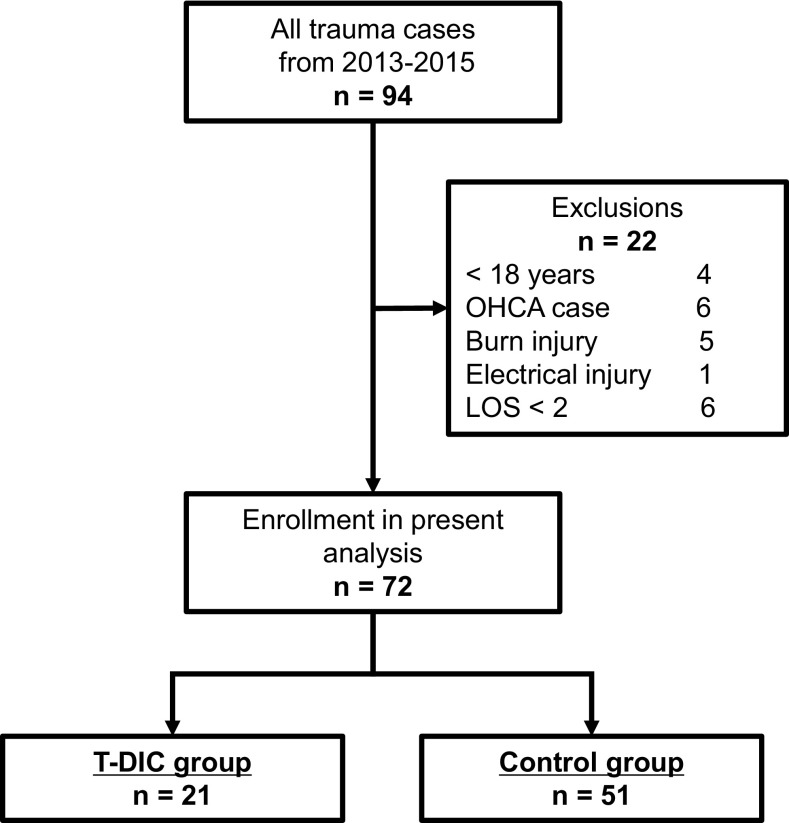
Study design

Ninety-four patients matched the inclusion criteria in this study (Fig. 2). However, 22 of them were eventually excluded: 4 for being less than 18 years old, 6 for out-of-hospital cardiac arrest, 5 for burn injury, 1 for electrical injury and 6 for less than 2 days of length of hospital stay. The 72 remaining patients were assigned to primary analysis. They were divided into two groups based on the presence of DIC on admission: traumatic-DIC (T-DIC) group (*n* = 21) and Control group (*n* = 51), respectively.

### Univariate analyses between the T-DIC group and the Control group

Table 1 shows patients’ characteristics, treatment, and clinical outcome. All were blunt trauma cases. The T-DIC group was significantly younger than the Control group (71 vs. 57 years; *p* = 0.034). Significantly more patients on warfarin were found in the T-DIC group compared with the Control group (14.3 vs. 0.0%; *p* = 0.022). The median JAAM DIC scores of both groups were 4 (IQR; 4–5) and 3 (0–3), respectively. No statistical differences were confirmed for sex, presence of shock, or other medical histories. According to trauma scales, the T-DIC group represented significantly higher ISS (29 vs. 12; *p* < 0.001), lower RTS (7.55 vs. 7.84; *p* = 0.001) and lower Ps (62.4 vs. 95.9; *p* = 0.001) compared with the Control group. In the T-DIC group, there were significantly more patients that received a massive blood transfusion within the first 24 h (42.9 vs. 5.9 %; *p* < 0.001), compared with the Control group. However, no association was found for emergency surgery and hospital mortality within both groups.

**Table 1:** Patients' characteristics, emergency treatment and clinical outcome in T-DIC and control groups

	T-DIC group (*n* = 21)	Control group (*n* = 51)	*p* values
Age, year, median (IQR)	71 (49–79)	57 (34–69)	0.034
Male, *n* (%)	13 (61.9)	34 (66.7)	0.700
Liver cirrhosis, *n* (%)	1 (4.8)	1 (2.0)	0.501
Antiplatelet agents, *n* (%)	3 (14.3)	3 (5.9)	0.233
Warfarin, *n* (%)	3 (14.3)	0 (0.0)	0.022
Other anticoagulants, *n* (%)	1 (4.8)	0 (0.0)	0.292
JAAM DIC score, pts, median (IQR)	4 (4–5)	3 (0–3)	<0.001
Shock, *n* (%)	4 (19.0)	2 (3.9)	0.056
Blunt trauma, *n* (%)	21 (100.0)	51 (100.0)	–
ISS, median (IQR)	29 (22–33)	12 (5–24)	<0.001
PTS, median (IQR)	7.55 (5.90–7.84)	7.84 (7.84–7.84)	0.001
Ps, %, median (IQR)	62.4 (41.5–91.0)	95.9 (83.6–98.4)	0.001
Massive transfusion, *n* (%)	9 (42.9)	3 (5.9)	<0.001
Emergency surgery, *n* (%)	7 (33.3)	10 (19.6)	0.173
Dead, *n* (%)	4 (19.0)	2 (3.9)	0.056

Laboratory tests are shown in Table 2. Complete blood counts in the T-DIC group demonstrated higher WBC (13,900 vs. 10,000; *p* = 0.007), lower Hb (10.8 vs. 13.5; *p* < 0.001) and lower PLT (15.5 vs. 22.0; *p* = 0.001) than the Control group. The same tendency was confirmed in the standard coagulation tests between the T-DIC group and the Control group: PT-INR (1.23 vs. 1.00; *p* < 0.001), APTT (35.4 vs. 28.9; *p* < 0.001), Fibrinogen (196 vs. 256; *p* = 0.012), FDP (149.9 vs. 30.9; *p* < 0.001) and DD (88.18 vs. 15.14; *p* < 0.001). Significantly higher lactate levels were associated with T-DIC compared with Control (3.2 vs. 1.8; *p* < 0.001).

**Table 2:** Laboratory tests in each group

	T-DIC group (*n* = 21)	Control group (*n* = 51)	*p* values
WBC, /µL, median (IQR)	13,900 (10,500–17,400)	10,000 (7700–12,600)	0.007
Hb, g/dL, median (IQR)	10.8 (9.0–12.1)	13.5 (12.3–15.2)	<0.001
PLT, 10^4^/µL, median (IQR)	15.5 (12.0–19.2)	22.0 (16.8–23.7)	0.001
PT-INR, median (IQR)	1.23 (1.13–1.58)	1.00 (0.96–1.05)	<0.001
APTT, second, median (IQR)	35.4 (31.1–44.5)	28.9 (26.5–32.2)	<0.001
Fibrinogen, mg/dL, median (IQR)	196 (103–276)	256 (204–299)	0.012
FDP, µg/mL, median (IQR)	149.9 (75.3–470.8)	30.9 (8.2–98.6)	<0.001
DD, µg/mL, median (IQR)	88.18 (38.78–251.85)	15.14 (4.67–51.7)	<0.001
Lactate, mmol/L, median (IQR)	3.2 (2.1–4.6)	1.8 (1.4–2.5)	<0.001

Thromboelastometric analyses revealed distinctive findings in T-DIC (Table 3). In the EXTEM test, T-DIC showed significantly longer CT (85 vs. 61; *p* = 0.002), lower A10 (50 vs. 54; *p* = 0.038), lower A20 (57 vs. 60; *p* = 0.048), higher LI30 (100 vs. 100; *p* = 0.030) and lower ML (10 vs. 15; *p* = 0.002) than the Control group. Consistent with the EXTEM test the INTEM test showed that significantly longer CFT (120 vs. 83; *p* = 0.003), lower α (67 vs. 73; *p* = 0.003), lower A10 (47 vs. 53; *p* = 0.002), lower A20 (54 vs. 59; *p* = 0.008), lower MCF (56 vs. 60; *p* = 0.019), higher LI30 (100 vs. 99; *p* = 0.002) and lower ML (8 vs. 14; *p* < 0.001), in the T-DIC group compared with the Control group. Other parameters, including the FIBTEM test, were not significantly different between the two groups. The ratio of hyperfibrinolysis was also equal in each group.

**Table 3:** Thromboelastometric analyses among groups

	T-DIC group (*n* = 21)		Control group (*n* = 51)		*p* values
EXTEM					
CT, s, median (IQR)	85	(61–118)	61	(50–70)	0.002
CFT, s, median (IQR)	109	(80–133)	93	(80–110)	0.155
α, ˚, median (IQR)	68	(65–74)	72	(69–75)	0.159
A10, mm, median (IQR)	50	(42–58)	54	(50–57)	0.038
A20, mm, median (IQR)	57	(51–64)	60	(57–63)	0.048
A30, mm, median (IQR)	59	(53–65)	61	(57–64)	0.136
MCF, mm, median (IQR)	59	(53–65)	61	(58–64)	0.168
LI30, %, median (IQR)	100	(100–100)	100	(99–100)	0.030
ML, %, median (IQR)	10	(6–14)	15	(11–17)	0.002
INTEM					
CT, s, median (IQR)	222	(192–298)	197	(156–271)	0.141
CFT, s, median (IQR)	120	(92–135)	83	(67–100)	0.003
α, ˚, median (IQR)	67	(64–72)	73	(71–77)	0.003
A10, mm, median (IQR)	47	(41–51)	53	(49–57)	0.002
A20, mm, median (IQR)	54	(49–58)	59	(55–62)	0.008
A30, mm, median (IQR)	57	(51–58)	59	(55–60)	0.058
MCF, mm, median (IQR)	56	(51–58)	60	(56–62)	0.019
LI30, %, median (IQR)	100	(100–100)	99	(99–100)	0.002
ML, %, median (IQR)	8	(5–11)	14	(10–18)	<0.001
FIBTEM					
MCF, mm, median (IQR)	10	(6–15)	13	(9–15)	0.135
Hyperfibrinolysis by ROTEM, *n* (%)	2	(9.5)	4/46	(8.7)	0.618

### The relationships between T-DIC and each parameter of JAAM DIC criteria

**Fig. 3:**
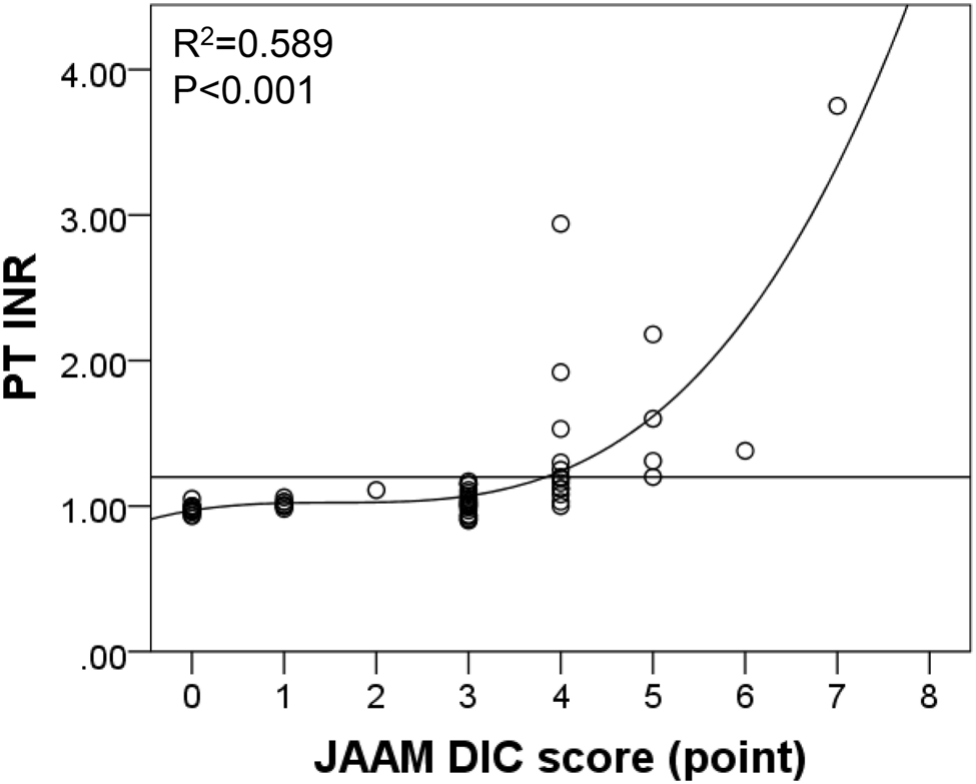
Spearman correlation analysis between PT-INR and JAAM DIC score

We evaluated the relationships between T-DIC and each parameter of the JAAM DIC criteria (Table 4). Almost all parameters, including SIRS ≥3 points (pts) (52.4 vs. 3.9%; *p* < 0.001), PLT <12 × 10^4^/µL (23.8 vs. 0.0%; *p* = 0.001), PT-INR ≥1.2 (60.0 vs. 0.0%; *p* < 0.001), FDP ≥25 µg/mL (100.0 vs. 54.8%; *p* < 0.001) and FDP ≥10 µg/mL (100.0 vs. 71.4 %; *p* = 0.005), were significantly associated with T-DIC. Next, we calculated the diagnostic accuracy for T-DIC by utilizing every parameter of the JAAM DIC criteria (Table 4). Higher sensitivity was observed in FDP ≥25 µg/mL (100.0%) and FDP ≥10 µg/mL (100.0%). In addition, higher specificity was observed for SIRS ≥3 pts (96.1%), PLT <8 × 10^4^/µL (100.0%), PLT <12 × 10^4^/µL (100.0 %) and PT-INR ≥1.2 (100.0%). Interestingly, PT-INR ≥1.2 showed the highest accuracy among all parameters. Moreover, Spearman correlation analysis revealed that PT-INR was statistically correlated with the JAAM DIC score more than other clinical scores (*r* = 0.709, *p* < 0.001). Curve fitting for the combination of PT-INR and the JAAM DIC score detected a significant non-linear association. The most obvious correlation was observed in the cubic curve-fitting equation (*R* ^*2*^ = 0.589, *p* < 0.001) (Fig. 3).

**Table 4:** The relationships between T-DIC and each parameter of JAAM DIC criteria

	Univariate analysis			Diagnostic accuracy for T-DIC				
	T-DIC group (*n* = 21)	Control group (*n* = 51)	*p* values	Sensitivity	Specificity	PPV	NPV	Accuracy
SIRS ≥3 pts, *n* (%)	11 (52.4)	2 (3.9)	<0.001	52.4	96.1	84.6	83.1	83.3
PLT <8 × 10^4^/µL, *n* (%)	1 (4.8)	0 (0.0)	0.292	4.8	100.0	100.0	71.8	72.2
PLT <12 × 10^4^/µL, *n* (%)	5 (23.8)	0 (0.0)	0.001	23.8	100.0	100.0	76.1	77.8
PT-INR ≥1.2, *n* (%)	12/20 (60.0)	0 (0.0)	<0.001	60.0	100.0	100.0	86.4	88.7
FDP ≥25 µg/mL, *n* (%)	20/20 (100.0)	23/42 (54.8)	<0.001	100.0	45.2	46.5	100.0	62.9
FDP ≥10 µg/mL, *n* (%)	20/20 (100.0)	30/42 (71.4)	0.005	100.0	28.6	40.0	100.0	51.6

### Thromboelastometric analysis of trauma induced coagulopathy based on 1.2 of PT-INR value

We divided patients into 2 groups based on their PT-INR value: PT-INR ≥1.2 group (*n* = 12) and PT-INR <1.2 group (*n* = 59), (Table 5). Univariate analyses were performed to evaluate the trauma-induced coagulopathy which was diagnosed by ROTEM in each group. In the EXTEM test, PT-INR ≥1.2 group showed statistically longer CT (99 vs. 61; *p* < 0.001), lower A10 (46 vs. 53; *p* = 0.034), lower A20 (54 vs. 60; *p* = 0.038) and lower ML (8 vs. 14; *p* = 0.006) than the PT-INR <1.2 group. Furthermore, significant differences were found in all parameters of the INTEM test as follows: CT (PT-INR ≥1.2 group: 231 vs. PT-INR <1.2 group: 197; *p* = 0.037), CFT (127 vs. 85; *p* = 0.012), α (66 vs. 73; *p* = 0.013), A10 (44 vs. 52; *p* = 0.001), A20 (52 vs. 58; *p* = 0.002), A30 (53 vs. 59; *p* = 0.010), MCF (54 vs. 59; *p* = 0.004), LI30 (100 vs. 100; *p* = 0.035) and ML (7 vs. 13; *p* = 0.002). In the FIBTEM test, a significantly lower MCF was confirmed in the PT-INR ≥1.2 group (8 vs. 13; *p* = 0.037). However, the percentage of patients with hyperfibrinolysis was not related to the PT-INR value.

**Table 5:** Thromboelastometric evaluations of trauma induced coagulopathy based on 1.2 of PT-INR value

	PT-INR ≥1.2 group (*n* = 12)	PT-INR <1.2 group (*n* = 59)	*p* values
EXTEM			
CT, s, median (IQR)	99 (74–128)	61 (50–74)	<0.001
CFT, s, median (IQR)	123 (80–134)	94 (80–110)	0.168
α, ˚, median (IQR)	66 (65–74)	72 (68–75)	0.159
A10, mm, median (IQR)	46 (38–56)	53 (49–59)	0.034
A20, mm, median (IQR)	54 (47–62)	60 (56–64)	0.038
A30, mm, median (IQR)	57 (49–63)	61 (56–64)	0.096
MCF, mm, median (IQR)	58 (50–63)	61 (57–65)	0.109
LI30, %, median (IQR)	100 (100–100)	100 (99–100)	0.052
ML, %, median (IQR)	8 (5–14)	14 (10–17)	0.006
INTEM			
CT, s, median (IQR)	231 (216–314)	197 (156–273)	0.037
CFT, s, median (IQR)	127 (97–157)	85 (69–119)	0.012
α, ˚, median (IQR)	66 (62–72)	73 (67–77)	0.013
A10, mm, median (IQR)	44 (38–49)	52 (48–57)	0.001
A20, mm, median (IQR)	52 (46–57)	58 (55–62)	0.002
A30, mm, median (IQR)	53 (48–58)	59 (54–60)	0.010
MCF, mm, median (IQR)	54 (48–58)	59 (56–62)	0.004
LI30, %, median (IQR)	100 (100–100)	100 (99–100)	0.035
ML, %, median (IQR)	7 (5–11)	13 (9–17)	0.002
FIBTEM			
MCF, mm, median (IQR)	8 (6–13)	13 (9–16)	0.037
Hyperfibrinolysis by ROTEM, *n* (%)	1 (8.3)	4 (7.4)	0.646

### The thromboelastometric parameters with significant differences evaluated by each parameter of the JAAM DIC criteria

**Fig. 4:**
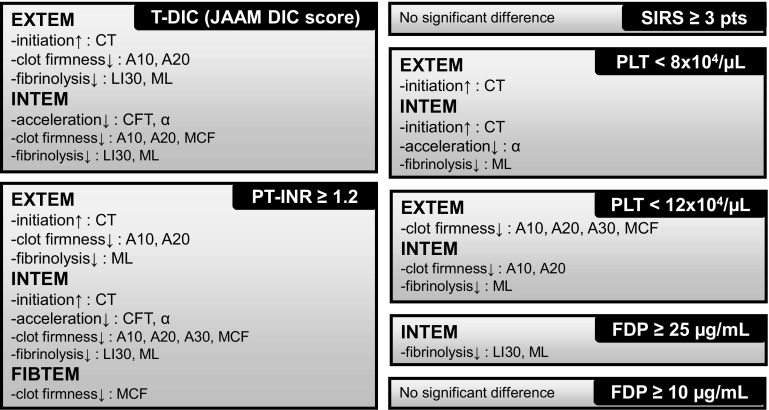
The thromboelastometric abnormalities associated with either T-DIC (JAAM DIC score ≥4 pts) or each parameter of the JAAM DIC criteria including PT-INR ≥1.2, SIRS ≥3 pts, PLT <8 × 104/µL, PLT <12 × 104/µL, FDP ≥25 µg/mL, FDP ≥10 µg/mL

We evaluated the correlation between ROTEM data and each parameter of the JAAM DIC criteria (Fig. 4). The left two panels (T-DIC and PT-INR ≥1.2) show results of the analyses described above. In addition, the same analyses were performed and results are shown in the 5 right-hand side panels (SIRS ≥3 pts, PLT <8 × 10^4^/µL, PLT <12 × 10^4^/µL, FDP ≥25 µg/mL, FDP ≥10 µg/mL) (detailed data not shown). The PT-INR of 1.2 was the most reliable detector for various kinds of coagulation and fibrinolytic abnormalities in trauma patients within all parameters of the JAAM DIC criteria.

## Discussion

This study demonstrates that a PT-INR of 1.2 is the most accurate diagnostic tool for T-DIC among the JAAM DIC criteria. The strong correlation between PT-INR ≥1.2 and the JAAM DIC score suggests that PT-INR is not only a reliable predictor, but is also a severity indicator for T-DIC. Furthermore, our results also enable many physicians working at community hospitals who have limited access to the data needed for the JAAM DIC criteria, to refer to PT-INR for detecting T-DIC.

PT-INR is a standard monitoring parameter for prophylaxis and thrombolytic therapy by anticoagulation agents that has been used for decades [23, 24]. There are also several evidences of this marker in trauma cases. An initial abnormality of PT on admission to a Level I trauma center is an independent risk factor for mortality (35% risk increase) [9]. Another retrospective cohort using more than 3000 trauma cases from five international hospitals concluded that the prothrombin time ratio >1.2 is a threshold level for higher mortality and transfusion requirements [11]. Our results also indicated that PT-INR could specifically detect the changes of trauma induced coagulopathy.

More and more reports about trauma-induced coagulopathy utilizing ROTEM have been published in this decade [19, 25, 26]. A recent retrospective study in Zurich reported that Hb ≥10 g/dL and abnormal MCF of INTEM were reliable predictors for massive transfusion in severely injured patients [25]. Another study in Salzburg concluded A10 and MCF of FIBTEM upon admission to the ED had high predictive value for massive transfusion [26]. The degree of hyperfibrinolysis diagnosed by ROTEM was correlated with clinical outcome, which was published by the same group in Salzburg [27]. ROTEM is able to separate the coagulation cascade into four main categories and patients with T-DIC showed these characteristic features in most categories of the EXTEM and INTEM tests in this study. Interestingly, the same tendency was found when the patients were divided by PT-INR ≥1.2 or <1.2. However, other parameters of the JAAM DIC criteria could not detect coagulopathy correctly compared with PT-INR. Considering these results, the viscoelastic testing might be able to reveal detailed differences in the coagulation and fibrinolytic status, whereas standard coagulation tests may fail to detect these differences.

This study has some limitations which will be addressed in future work. First, this is a retrospective study and a small sample size may cause a selection bias. Second, since trauma patients tend to have multiple medical conditions with various severities, the coagulation and fibrinolytic status of trauma patients drastically changes during their clinical course [28]. Finally, we could not control for medication use as a potential confounding factor, which may affect PT-INR values. There were significantly more patients on warfarin in the T-DIC group and higher PT-INR group (data not shown) than others.

This study reveals a tendency to diagnose T-DIC with respect to each parameter of the JAAM DIC criteria. Among the parameters, higher specificity was confirmed in the subject of SIRS ≥3 pts, PLT <8 × 10^4^/µL, PLT <12 × 10^4^/µL, and PT-INR ≥1.2 whereas higher sensitivity was observed in FDP ≥25 µg/mL and FDP ≥10 µg/mL. This means that SIRS, PLT and PT-INR are useful parameters to make the diagnosis of T-DIC, while FDP is informative to rule out the diagnosis. Most importantly, if we encounter a severe trauma patient with high PT-INR, the patient is highly likely to present DIC and immediate curative interventions should be considered.

## Conclusions

Among the JAAM DIC criteria, the PT-INR ≥1.2 possesses the highest accuracy for the diagnosis of trauma-induced DIC and for an evaluation of its severity utilizing thromboelastometry.
